# Supplementary material for: The skeletal muscle circadian clock regulates titin splicing through RBM20
Source: eLife. 2022 Sep 1;11:e76478. doi: 10.7554/eLife.76478 (PMC9473687; doi:10.7554/eLife.76478)

Figure 1-Source Data 1

1. Global *Bmal1* KO LV
2. TA iMS*Bmal1*$^{+/+}$ - M47
3. TA iMS*Bmal1*$^{+/+}$ - M90
4. TA iMS*Bmal1*$^{+/+}$ - M111
5. TA iMS*Bmal1*$^{+/+}$ - M114
6. TA iMS*Bmal1*$^{-/-}$ - M61
7. TA iMS*Bmal1*$^{-/-}$ - M62
8. TA iMS*Bmal1*$^{-/-}$ - M76
9. TA iMS*Bmal1*$^{-/-}$ - M77
10 - 13. Not relevant to this project.
14. WT LV
15. WT LV

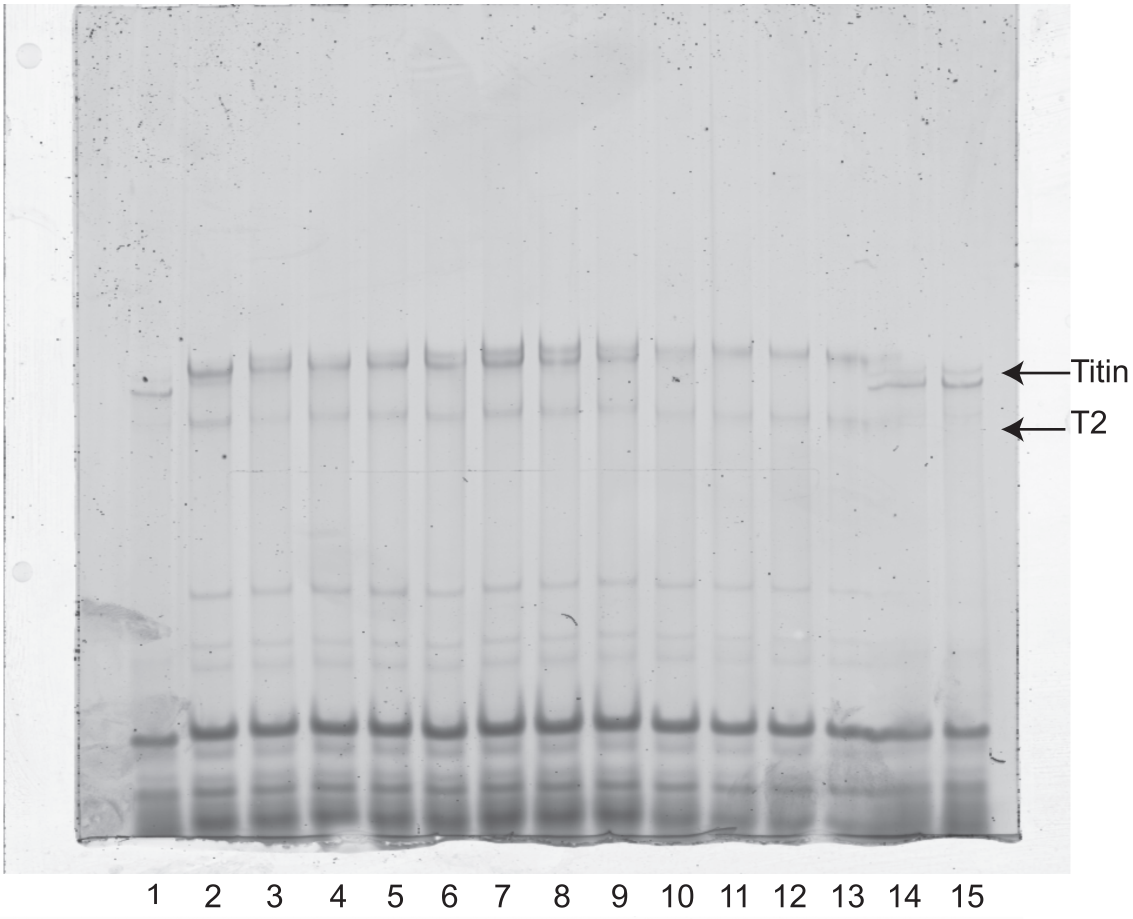

1. WT GTN
2. WT TA
3. WT GTN
4. WT LV
5. WT TA
6. EDL iMS*Bmal1*$^{+/+}$ - M1
7. EDL iMS*Bmal1*$^{-/-}$ - M7
8. TA iMS*Bmal1*$^{-/-}$ - M1
9. TA iMS*Bmal1*$^{-/-}$ - M7
10 - 13. Not relevant to this project.
14. WT LV
15. WT TA

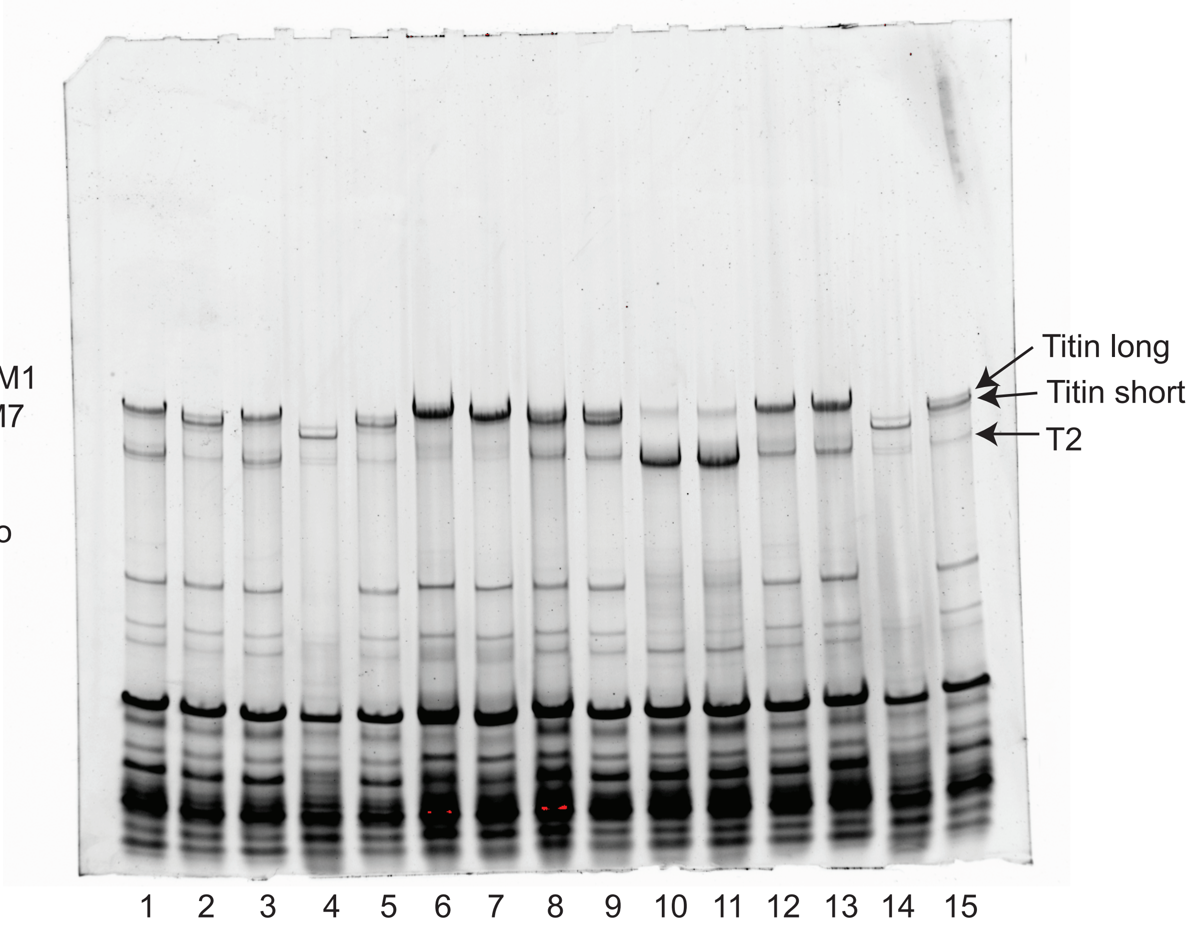

Supplement: Figure 1—source data 1. [file elife-76478-fig1-data1.zip › Figure 1-source data 1/Figure 1-source data 1.pdf]
