# Supplementary figures and images for: The skeletal muscle circadian clock regulates titin splicing through RBM20
Source: eLife. 2022 Sep 1;11:e76478. doi: 10.7554/eLife.76478 (PMC9473687; doi:10.7554/eLife.76478)

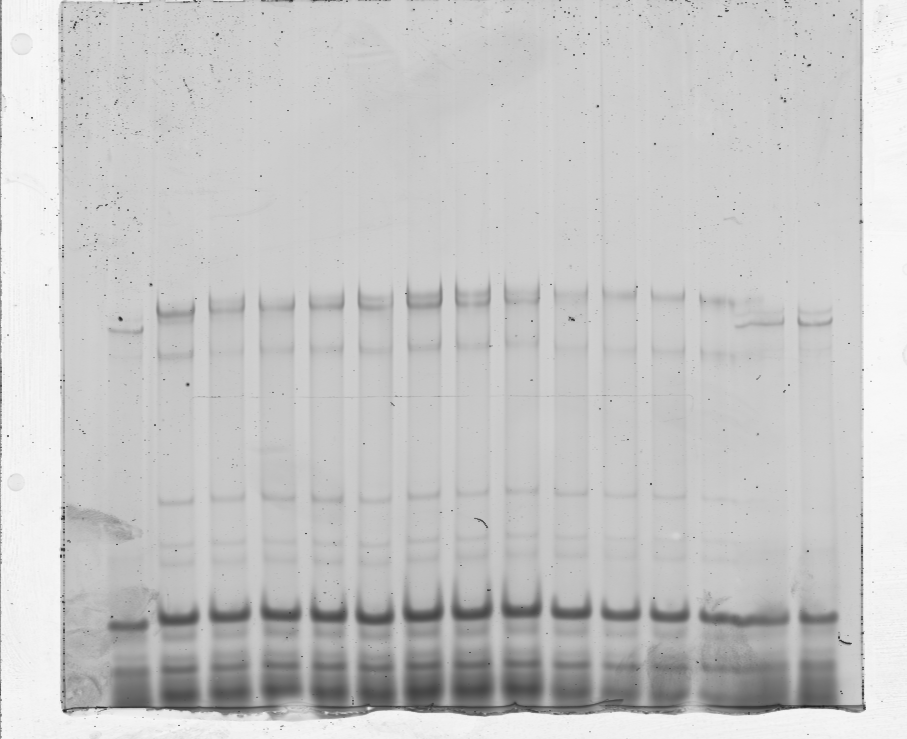

Supplement: Figure 1—source data 1. [file elife-76478-fig1-data1.zip › Figure 1-source data 1/Figure 1-source data 1-Titin 1.tif]

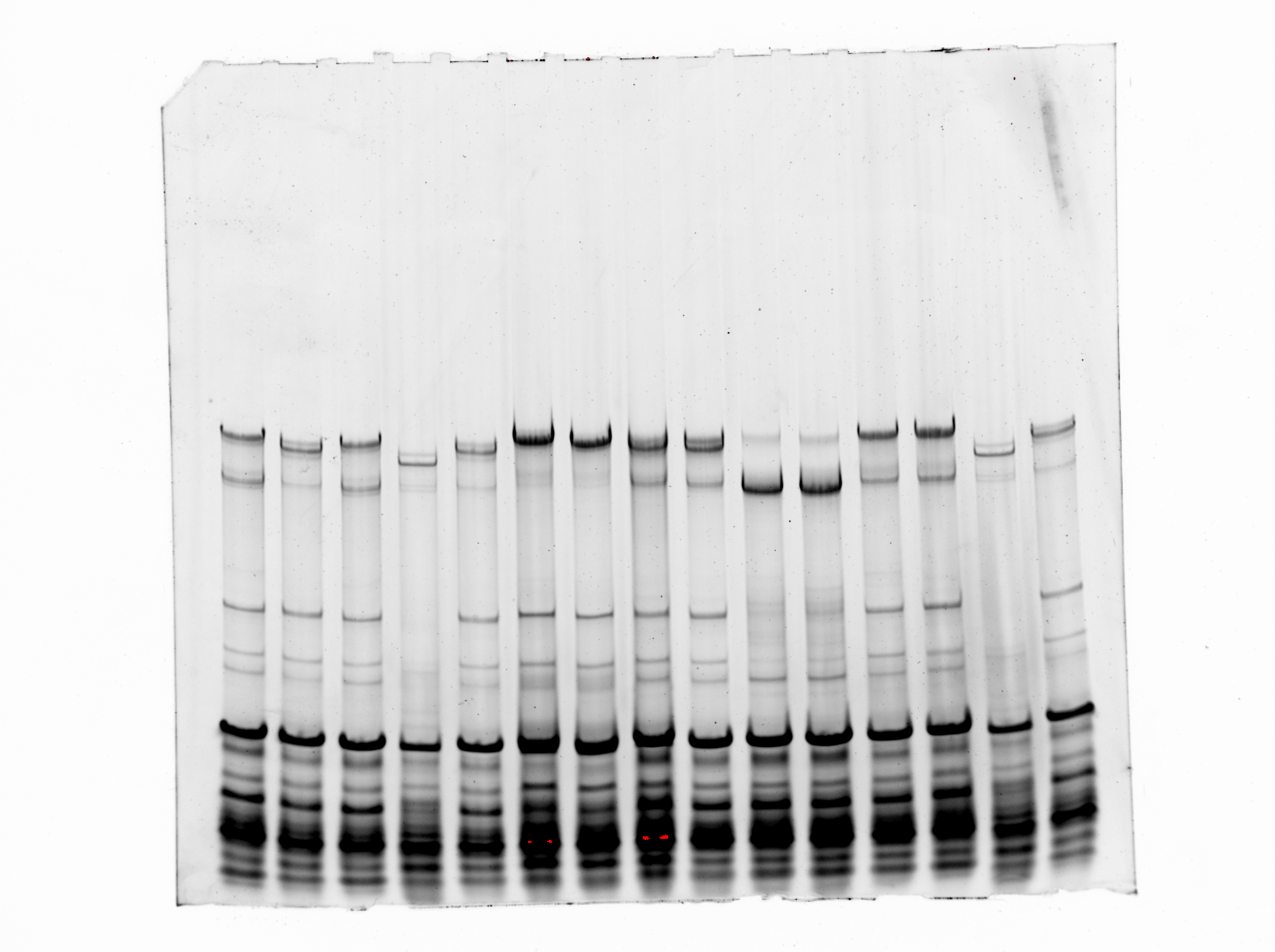

Supplement: Figure 1—source data 1. [file elife-76478-fig1-data1.zip › Figure 1-source data 1/Figure 1-source data 1-Titin 2.tif]

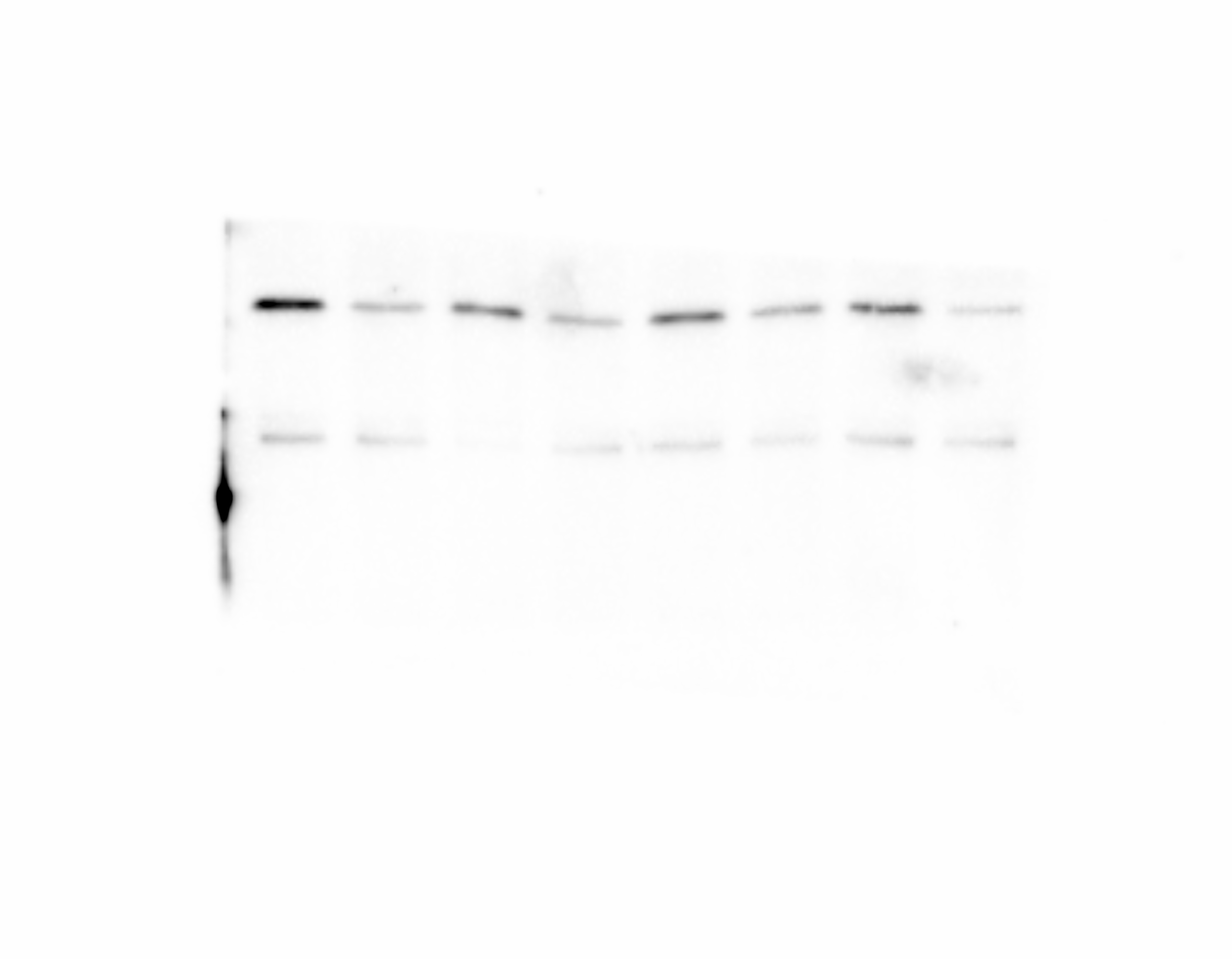

Supplement: Figure 5—source data 1. — Western blot of alternating lanes of iMSBmal1+/+ and iMSBmal1-/- muscle lysates probed with anti-RBM20 antibody (top). Western blot of alternating lanes of iMSBmal1+/+ and iMSBmal1-/- muscle lysates probed with anti-γ-tubulin antibody (bottom). [file elife-76478-fig5-data1.zip › Figure 5-source data 1/Figure 5-source data 1-RBM20.tif]

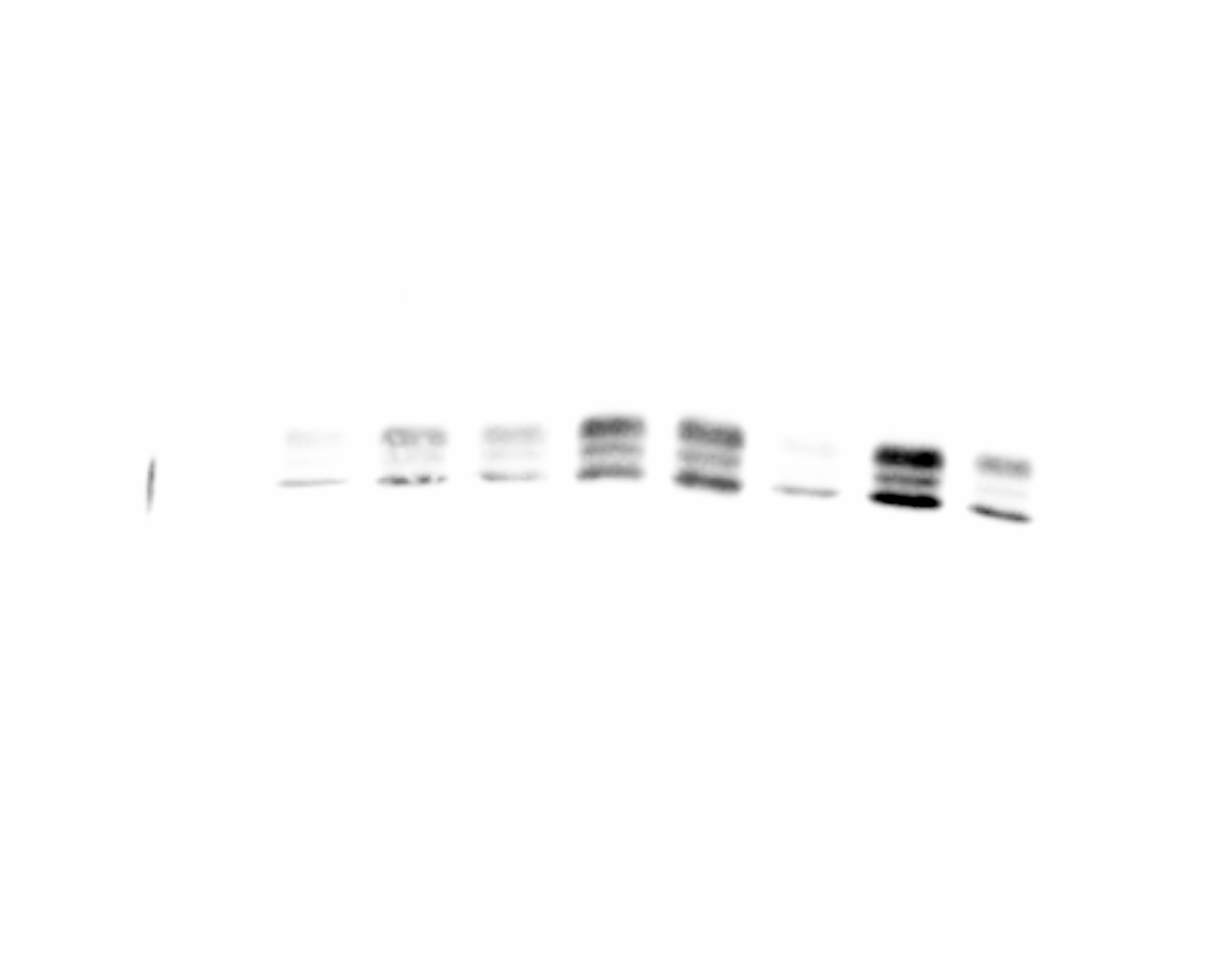

Supplement: Figure 5—source data 1. — Western blot of alternating lanes of iMSBmal1+/+ and iMSBmal1-/- muscle lysates probed with anti-RBM20 antibody (top). Western blot of alternating lanes of iMSBmal1+/+ and iMSBmal1-/- muscle lysates probed with anti-γ-tubulin antibody (bottom). [file elife-76478-fig5-data1.zip › Figure 5-source data 1/Figure 5-source data 1-Tubulin.tif]

Figure 5 - Source Data 1

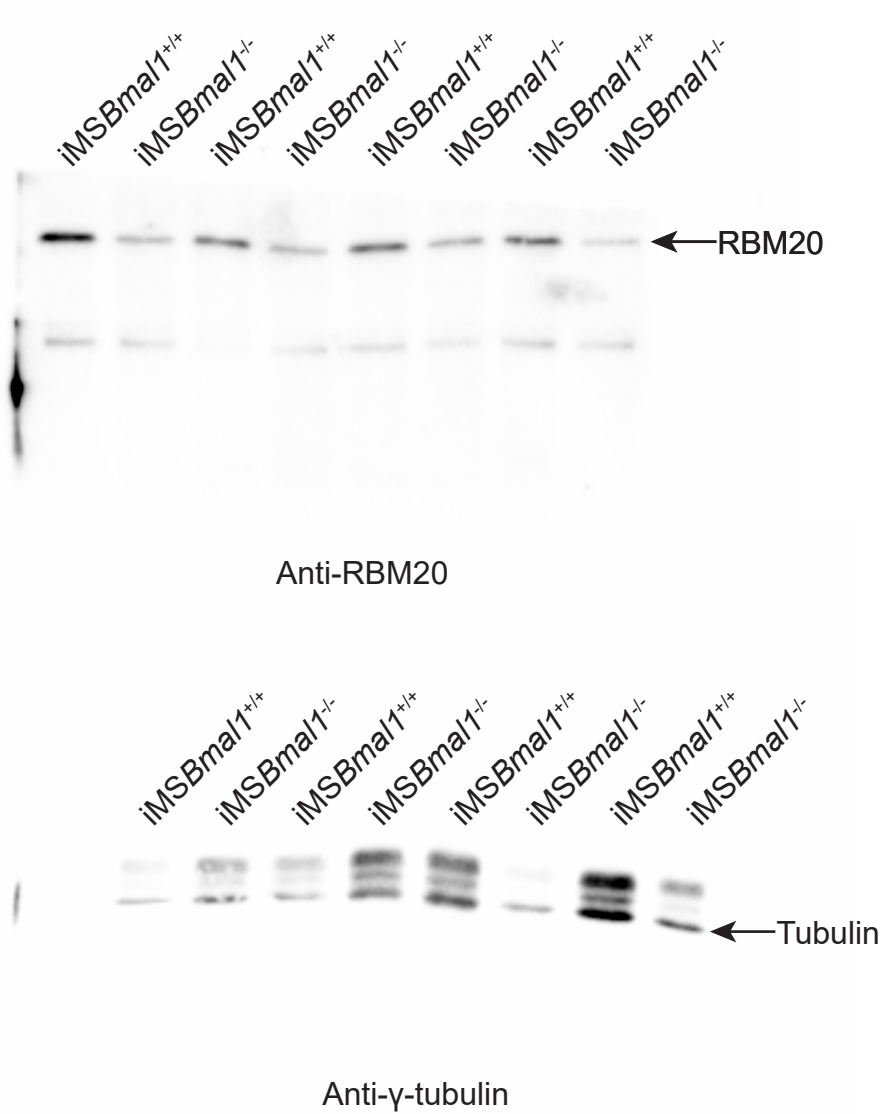

Supplement: Figure 5—source data 1. — Western blot of alternating lanes of iMSBmal1+/+ and iMSBmal1-/- muscle lysates probed with anti-RBM20 antibody (top). Western blot of alternating lanes of iMSBmal1+/+ and iMSBmal1-/- muscle lysates probed with anti-γ-tubulin antibody (bottom). [file elife-76478-fig5-data1.zip › Figure 5-source data 1/Figure 5-source data 1.pdf]

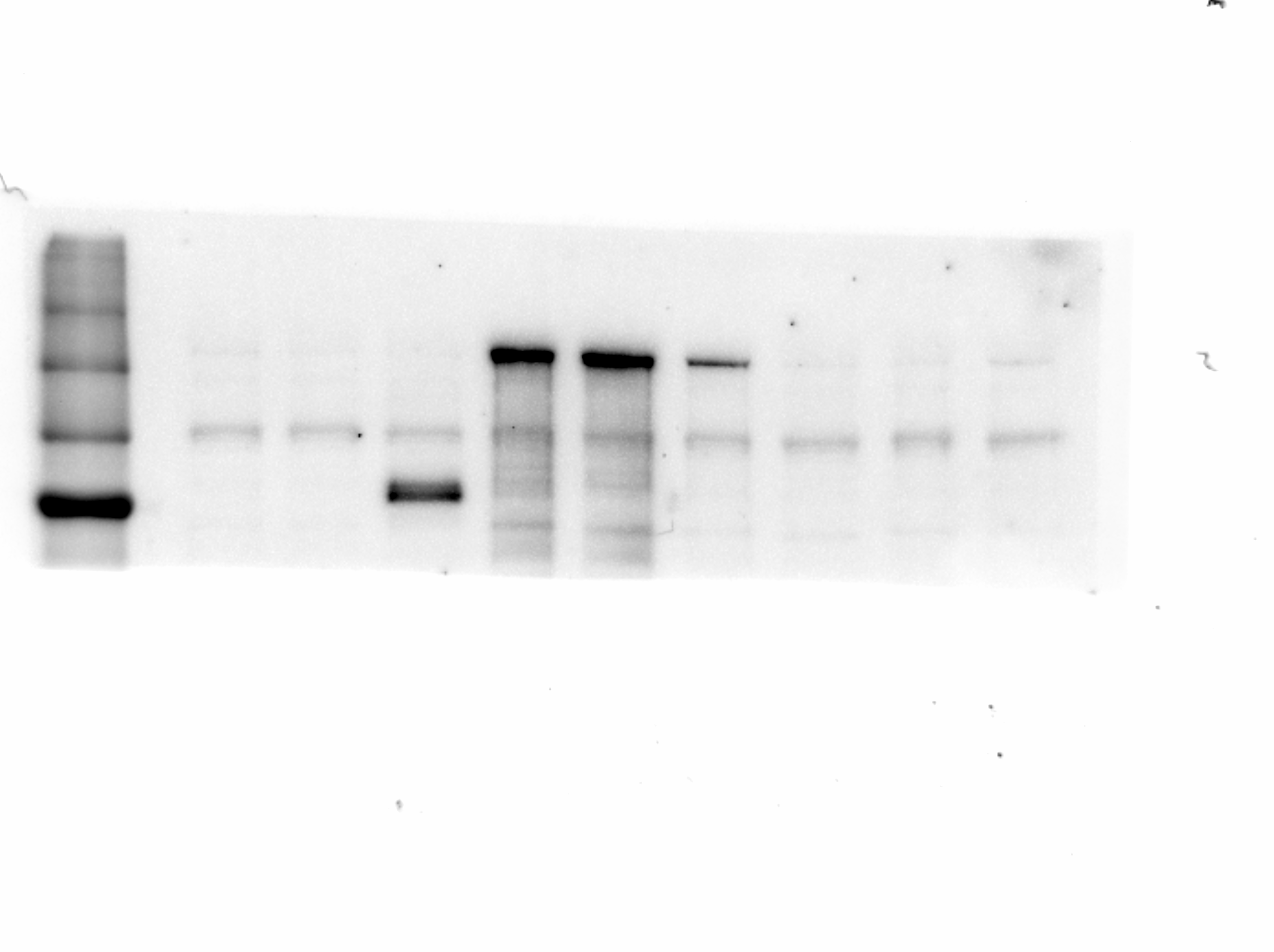

Supplement: Figure 6—source data 1. — Western blot of HA protein expression across groups (top-left). Western blot of γ-tubulin expression across groups (top-right). Western blot of RBM20 expression across groups (middle left). Western blot of γ-tubulin expression across groups (middle right). Rescue of RBM20 expression results in titin isoform ratio similar to isoform ratio in wildtype muscle (bottom). [file elife-76478-fig6-data1.zip › Figure 6-source data 1/Figure 6-source data 1-HA.tif]

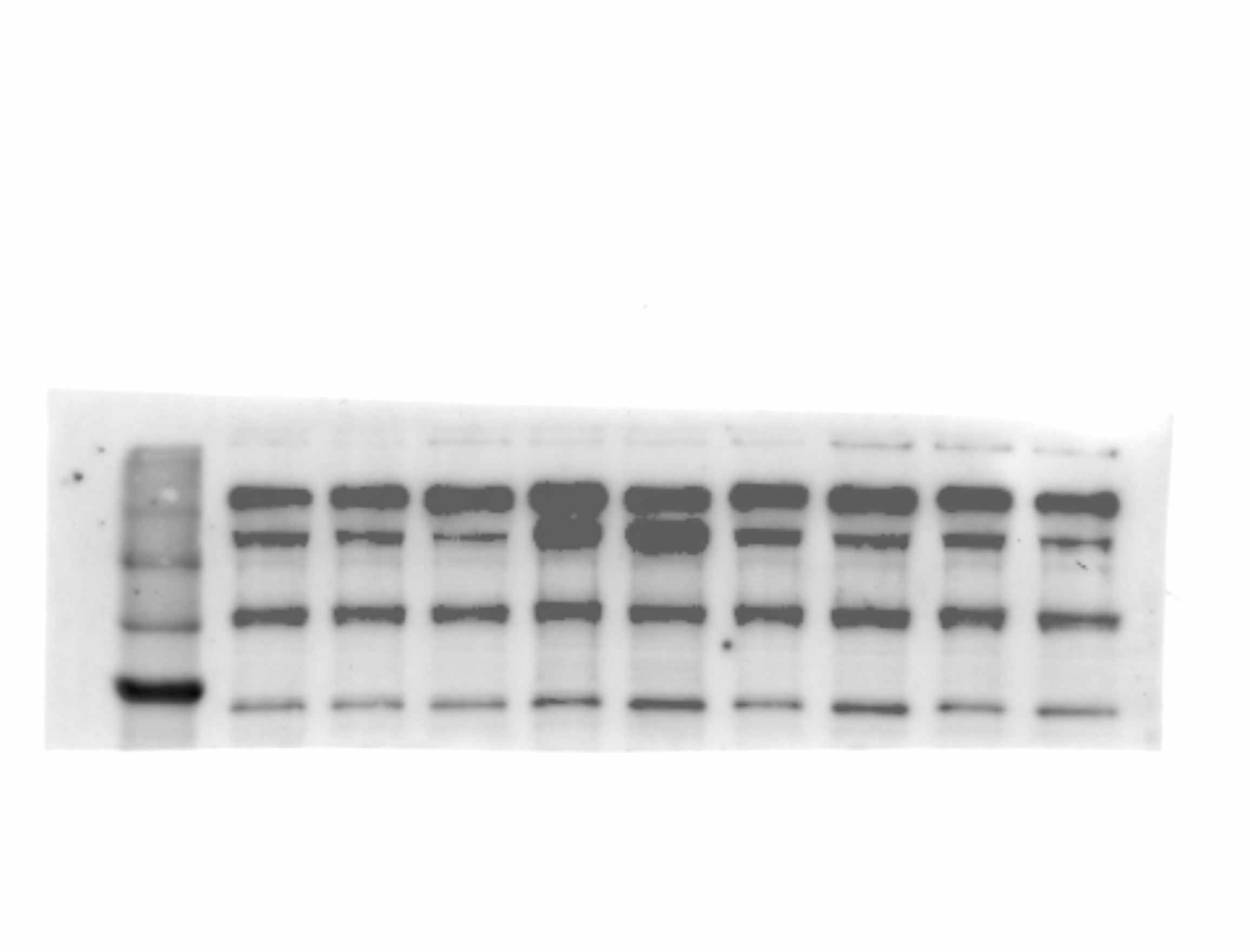

Supplement: Figure 6—source data 1. — Western blot of HA protein expression across groups (top-left). Western blot of γ-tubulin expression across groups (top-right). Western blot of RBM20 expression across groups (middle left). Western blot of γ-tubulin expression across groups (middle right). Rescue of RBM20 expression results in titin isoform ratio similar to isoform ratio in wildtype muscle (bottom). [file elife-76478-fig6-data1.zip › Figure 6-source data 1/Figure 6-source data 1-RBM20.tif]

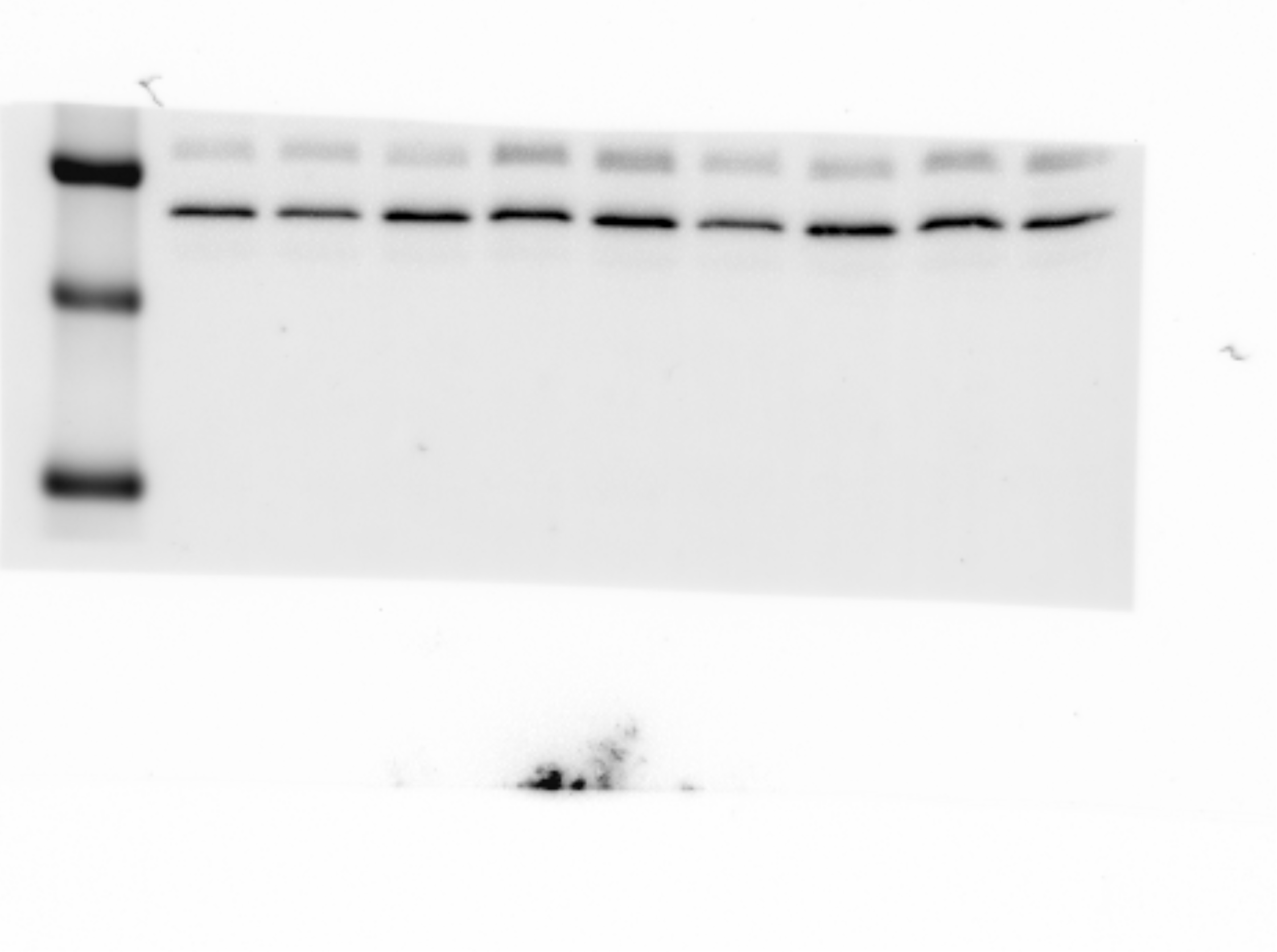

Supplement: Figure 6—source data 1. — Western blot of HA protein expression across groups (top-left). Western blot of γ-tubulin expression across groups (top-right). Western blot of RBM20 expression across groups (middle left). Western blot of γ-tubulin expression across groups (middle right). Rescue of RBM20 expression results in titin isoform ratio similar to isoform ratio in wildtype muscle (bottom). [file elife-76478-fig6-data1.zip › Figure 6-source data 1/Figure 6-source data 1-tubulin for HA.tif]

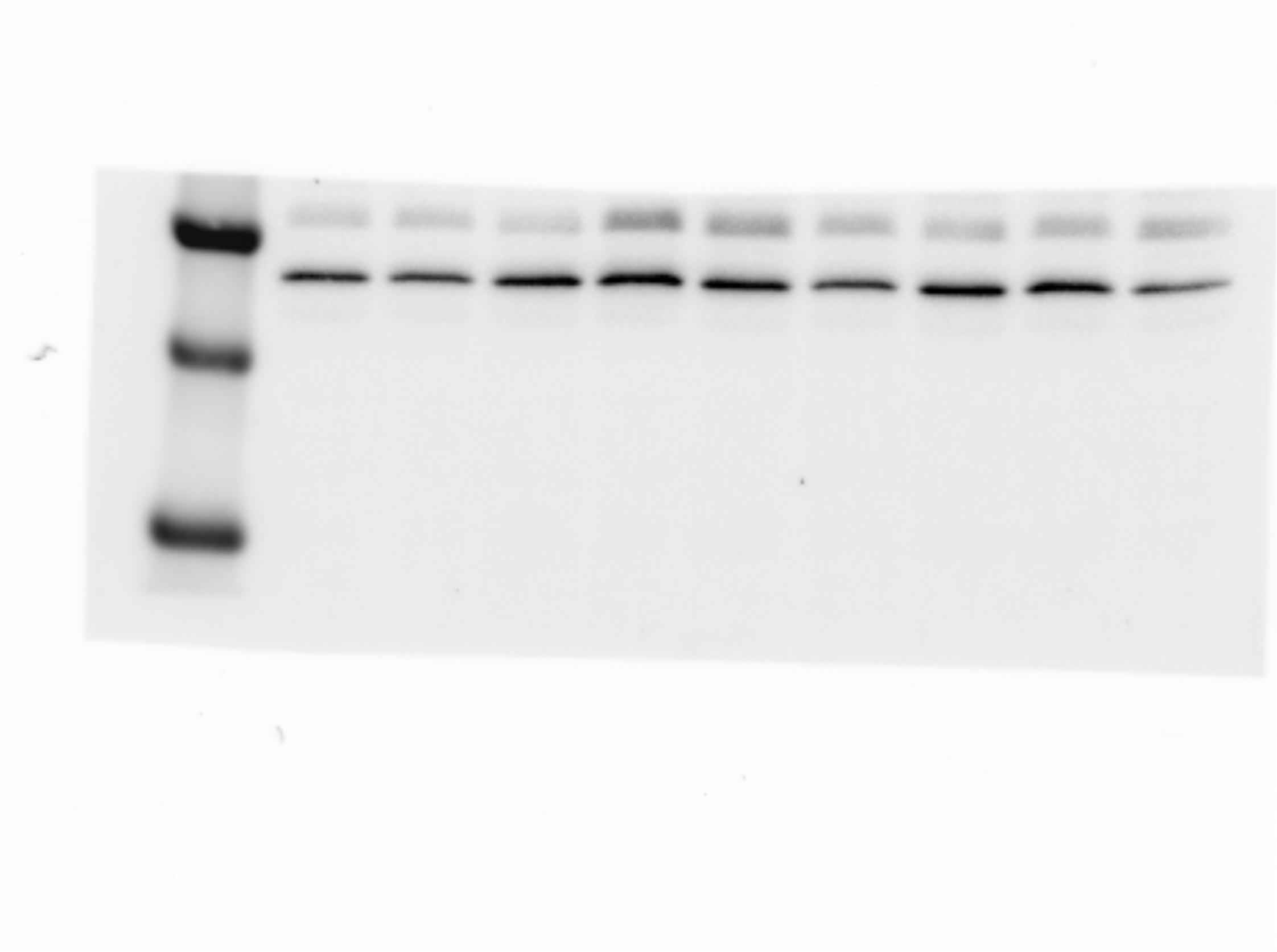

Supplement: Figure 6—source data 1. — Western blot of HA protein expression across groups (top-left). Western blot of γ-tubulin expression across groups (top-right). Western blot of RBM20 expression across groups (middle left). Western blot of γ-tubulin expression across groups (middle right). Rescue of RBM20 expression results in titin isoform ratio similar to isoform ratio in wildtype muscle (bottom). [file elife-76478-fig6-data1.zip › Figure 6-source data 1/Figure 6-source data 1-tubulin for RBM20.tif]
