# Supplementary material for: The skeletal muscle circadian clock regulates titin splicing through RBM20
Source: eLife. 2022 Sep 1;11:e76478. doi: 10.7554/eLife.76478 (PMC9473687; doi:10.7554/eLife.76478)

Figure 6 - Source Data 1

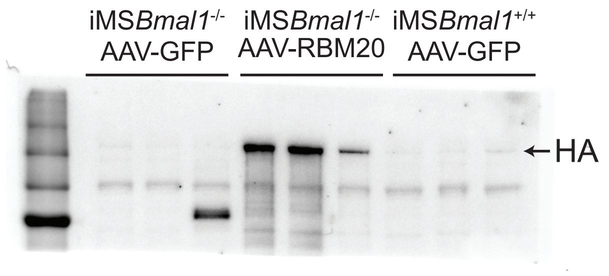

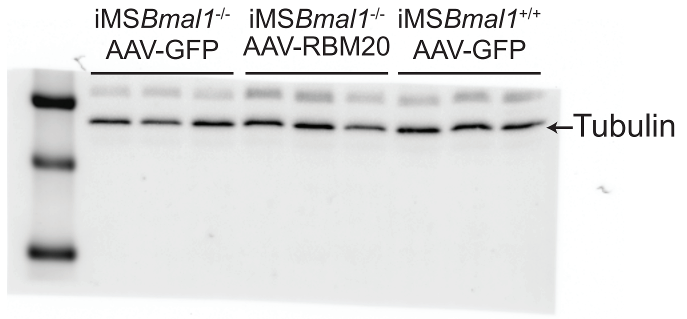

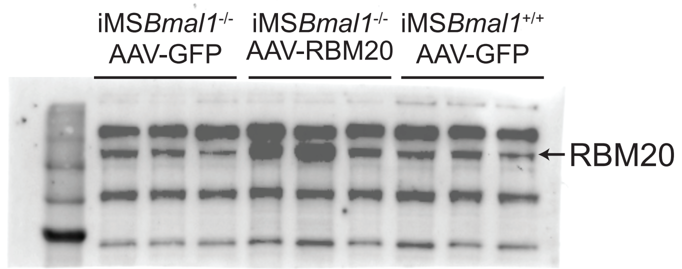

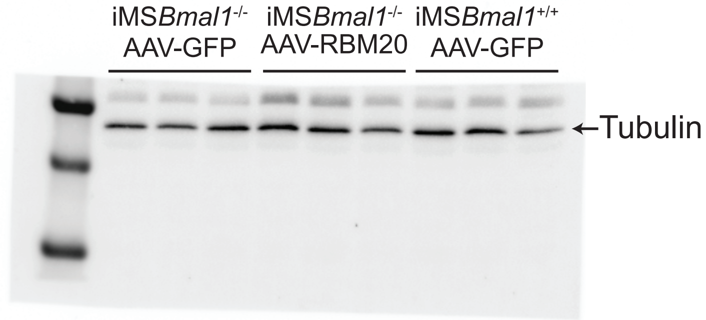

1,447 L TAM Bmal1 HA
2, heart
3, 243 R VEH
4, 441 R TAM GFP
5, 262 L TAM Rbm20 HA
6, 238 R VEH
7, 443 R TAM GFP
8, 637 L TAM Rbm20 HA
9, 243 L VEH
10, 262 R TAM GFP
11, 639 L TAM Rbm20 HA
12, 259 L VEH
13, 249R TAM GFP
14, 249 L TAM Rbm20 HA
15, 441 L TAM Bmal1 HA

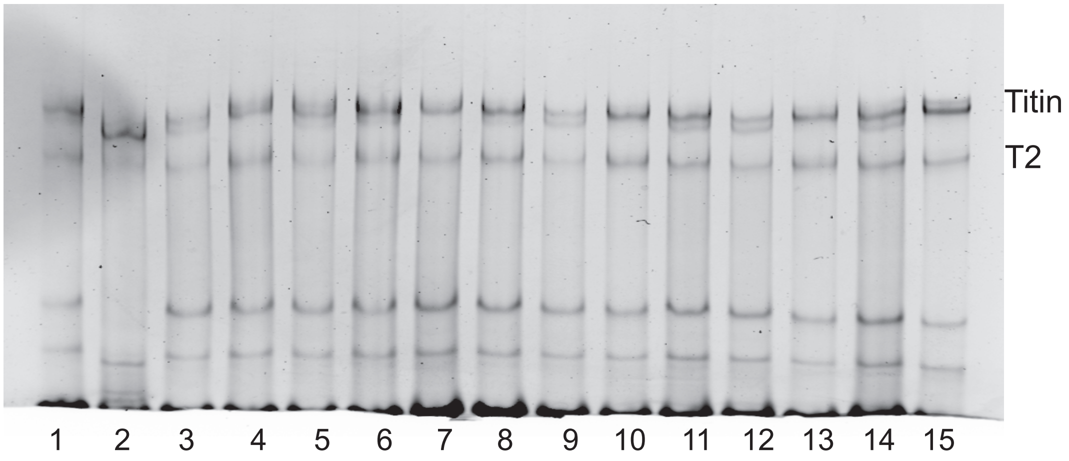

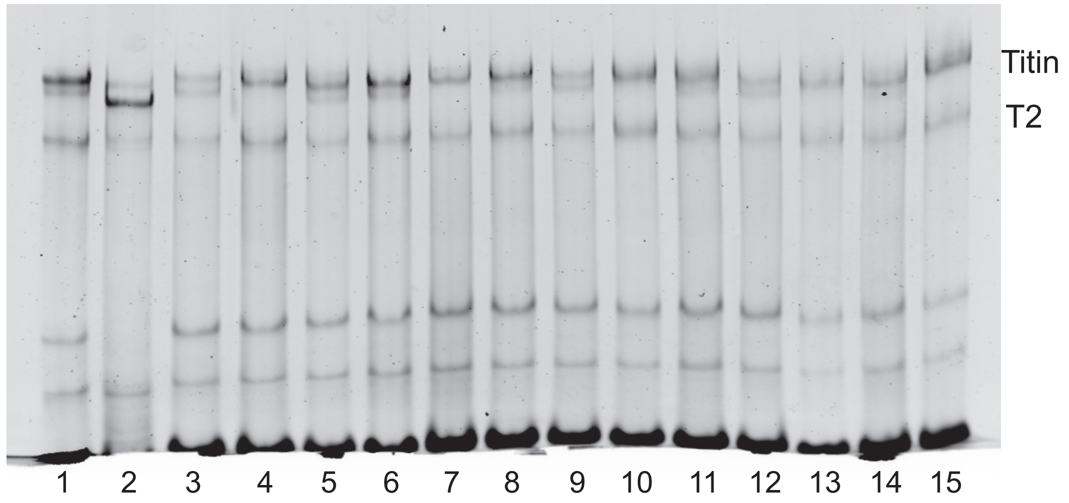

Supplement: Figure 6—source data 1. — Western blot of HA protein expression across groups (top-left). Western blot of γ-tubulin expression across groups (top-right). Western blot of RBM20 expression across groups (middle left). Western blot of γ-tubulin expression across groups (middle right). Rescue of RBM20 expression results in titin isoform ratio similar to isoform ratio in wildtype muscle (bottom). [file elife-76478-fig6-data1.zip › Figure 6-source data 1/Figure 6-source data 1.pdf]
