# Supplementary material for: The skeletal muscle circadian clock regulates titin splicing through RBM20
Source: eLife. 2022 Sep 1;11:e76478. doi: 10.7554/eLife.76478 (PMC9473687; doi:10.7554/eLife.76478)
Supplement: Transparent reporting form [file elife-76478-transrepform1.docx]

***eLife’s* transparent reporting form**

We encourage authors to provide detailed information *within their submission* to facilitate the interpretation and replication of experiments. Authors can upload supporting documentation to indicate the use of appropriate reporting guidelines for health-related research (see [EQUATOR Network](http://www.equator-network.org/%20)), life science research (see the [BioSharing Information Resource](https://biosharing.org/)), or the [ARRIVE guidelines](http://www.plosbiology.org/article/info:doi/10.1371/journal.pbio.1000412) for reporting work involving animal research. Where applicable, authors should refer to any relevant reporting standards documents in this form.

If you have any questions, please consult our Journal Policies and/or contact us: [editorial@elifesciences.org](mailto:editorial@elifesciences.org).

**Sample-size estimation**

- You should state whether an appropriate sample size was computed when the study was being designed
- You should state the statistical method of sample size computation and any required assumptions
- If no explicit power analysis was used, you should describe how you decided what sample (replicate) size (number) to use

Please outline where this information can be found within the submission (e.g., sections or figure legends), or explain why this information doesn’t apply to your submission:

Sample sizes were calculated using computational simulations by our late colleague Dr. Michael Hughes (Washington University of St. Louis) to determine the sample size needed for reliable detection of titin isoform differences.

**Replicates**

- You should report how often each experiment was performed
- You should include a definition of biological versus technical replication
- The data obtained should be provided and sufficient information should be provided to indicate the number of independent biological and/or technical replicates
- If you encountered any outliers, you should describe how these were handled
- Criteria for exclusion/inclusion of data should be clearly stated
- High-throughput sequence data should be uploaded before submission, with a private link for reviewers provided (these are available from both GEO and ArrayExpress)

Please outline where this information can be found within the submission (e.g., sections or figure legends), or explain why this information doesn’t apply to your submission:

For our experiments, biological replicates are individual mice or a separate vial of C2C12-GFP cells. Technical replicates are separate sections of muscle from the same mice or cells originating from the same vial of cells. Biological replicates are reported within figure legends and technical replicates are reported in methods where applicable. For high-throughput sequencing (Figures 1 and 6), data have been deposited to GEO under the accession number GSE189865 (reviewer access token chktmoicrhkxhgn). C2C12-GFP cells were authenticated as part of a concurrent experiment whose data have been deposited to GEO under the accession number GSE148667.

**Statistical reporting**

- Statistical analysis methods should be described and justified
- Raw data should be presented in figures whenever informative to do so (typically when N per group is less than 10)
- For each experiment, you should identify the statistical tests used, exact values of N, definitions of center, methods of multiple test correction, and dispersion and precision measures (e.g., mean, median, SD, SEM, confidence intervals; and, for the major substantive results, a measure of effect size (e.g., Pearson's r, Cohen's d)
- Report exact p-values wherever possible alongside the summary statistics and 95% confidence intervals. These should be reported for all key questions and not only when the p-value is less than 0.05.

Please outline where this information can be found within the submission (e.g., sections or figure legends), or explain why this information doesn’t apply to your submission:

Statistical methods are reported as the mean ± SEM. Statistical tests used are outlined in the methods section.

(For large datasets, or papers with a very large number of statistical tests, you may upload a single table file with tests, Ns, etc., with reference to sections in the manuscript.)

**Group allocation**

- Indicate how samples were allocated into experimental groups (in the case of clinical studies, please specify allocation to treatment method); if randomization was used, please also state if restricted randomization was applied
- Indicate if masking was used during group allocation, data collection and/or data analysis

Please outline where this information can be found within the submission (e.g., sections or figure legends), or explain why this information doesn’t apply to your submission:

Mice within cages were randomly assigned to either experimental or control conditions. Masking was used when confirming RNAseq data using LC-MS, during imaging, and during image analysis.

**Additional data files (“source data”)**

- We encourage you to upload relevant additional data files, such as numerical data that are represented as a graph in a figure, or as a summary table
- Where provided, these should be in the most useful format, and they can be uploaded as “Source data” files linked to a main figure or table
- Include model definition files including the full list of parameters used
- Include code used for data analysis (e.g., R, MatLab)
- Avoid stating that data files are “available upon request”

Please indicate the figures or tables for which source data files have been provided:

All code used is standard R code using standard parameters. All R packages used are highlighted in the methods section. Figure 1-Source Data 1 is the SDS-VAGE gels used for quantifying titin isoform ratios. Figure 1-Source Data 2 is provided as this is the LC-MS data used to calculate titin splicing at the protein level. Figure 5-Source Data 1 is uncut western blots used for measuring RBM20 expression. Figure 6-Source Data 1 is uncut western blots used for measuring RBM20 expression and SDS-VAGE gels used for measuring titin isoform ratios. Figure 6-Figure Supplement 1 is representative images and quantification of images used for measuring sarcomere lengths.
